# Mobility-informed hospital siting for accessible and lower-carbon healthcare travel: an ensemble learning study in Tianjin, China

**DOI:** 10.3389/fpubh.2026.1934861

**Published:** 2026-09-14

**Authors:** Jianxun Zhang, Fangfei Liu, Lei Wang, Huanjuan Yang, Rui Zhu, Chengying Li

**Affiliations:** 1 School of Civil Engineering and Water Resources, Qinghai University, Xining, China; 2 Key Laboratory of Energy-Saving Building Materials and Engineering Safety, Xining, Qinghai, China; 3 Key Laboratory of Water Ecology Remediation and Protection at Headwater Regions of Big Rivers, Ministry of Water Resources, Xining, China; 4 Laboratory of Ecological Protection and High-Quality Development in the Upper Yellow River, Xining, Qinghai, China; 5 School of Earth and Space Sciences, Peking University, Beijing, China; 6 Qinghai Provincial People's Hospital, Xining, China; 7 Warwick Manufacturing Group (WMG), University of Warwick, Coventry, United Kingdom

**Keywords:** ensemble learning, healthcare accessibility, healthcare travel behavior, hospital siting, low-carbon mobility, urban public health

## Abstract

Healthcare facility layouts shape access to care, travel burdens, and travel-related carbon emissions. Conventional siting assessments, however, often rely on supply-side indicators without accounting for how patients actually travel. This study examined nine tertiary Grade-A hospitals in central Tianjin using 2,596 questionnaires from hospital visitors and spatial data on road networks, bus and metro stations, expressways, and points of interest. Six spatial and transport indicators were evaluated: surveyed medical-visitor density, regional service coverage, transport accessibility, distance to expressways, public transit coverage, and road network density. An ensemble framework dominated by a graph convolutional network was used to predict supply-demand relationships between respondents and hospitals and to compare scenarios in terms of service utility, travel efficiency, and lower-carbon performance. Respondents generally reported a travel tolerance of within 20 km and 60 min, while their willingness to use direct public transport declined as the number of stops increased. The ensemble achieved a reported validation accuracy above 90% under the study’s validation procedure. Among the scenarios evaluated, J1 combined medium surveyed medical-visitor density, high service coverage, high accessibility, greater distance from expressways, high public transit coverage, and medium road network density. This scenario had the lowest relative carbon-emission level. By incorporating observed mobility constraints, the framework may support more accessible and lower-carbon planning of urban public-health infrastructure. Because the analysis uses a hospital-based survey from one city, broader application requires population-based data and external validation.

## 1 Introduction

The shortage and uneven spatial distribution of high-quality medical resources remain structural challenges worldwide. The Organization for Economic Co-operation and Development has reported that shortages of healthcare professionals and facilities can create unequal physical access to care (1). In China, 8% of cities function as national or regional healthcare hubs, whereas 35% face healthcare shortages and difficulties in meeting local healthcare needs (2). In Surrey, Canada, only 56.2% of residents could reach at least one healthcare facility within 30 min by public transport (3). Spatial mismatches can lengthen healthcare trips, concentrate travel demand in urban centers, and increase congestion around hospitals. Urban traffic further compounds the environmental burden. Peak-hour vehicle emissions can be 4.51 times those under normal traffic conditions (4), while congestion can increase the external costs of air pollutants and greenhouse gases by approximately 50% relative to free flow (5). Hospital siting should therefore consider healthcare access, travel efficiency, and travel-related emissions together.

Limited spatial access to healthcare disproportionately affects vulnerable groups and can hinder inclusive urban development (3). The inclusive-city concept promoted by the United Nations Human Settlements Programme emphasizes access to urban opportunities across population groups (6). Hospital siting and accessibility studies, however, often use supply-oriented indicators such as population demand, facility capacity, service areas, distance, and travel time (7, 8). These measures may equate potential service coverage with actual access and overlook differences in patient travel behavior (8–10). In practice, reaching a hospital also depends on travel-time tolerance, transfer burden, and available transport modes (11–13). These constraints are particularly important for patients who are physically unwell, time constrained, or mobility limited.

Incorporating observed healthcare travel behavior can address an important limitation of supply-oriented hospital siting. Patient choices are heterogeneous, and the associations of distance and travel time with hospital choice may be nonlinear (14, 15). Similar context dependence has been reported in built-environment studies (16). A behavior-informed assessment must therefore integrate survey variables with road networks, transit stations, expressways, and time-varying traffic data (17–19). These sources differ in scale, format, and update frequency. Linking them to large survey datasets also increases computational demands. An appropriate framework must accommodate heterogeneous spatial and behavioral data, graph-structured relationships, and nonlinear interactions.

This study examines nine tertiary Grade-A hospitals in central Tianjin using 2,596 questionnaires from surveyed hospital visitors and multi-source spatial data. The analysis uses six indicators: surveyed medical-visitor density, regional service coverage, transport accessibility, distance to expressways, public transit coverage, and road network density. A graph convolutional network (GCN)-dominant ensemble framework predicts respondent-hospital supply–demand relationships. The prediction outputs are combined with the study’s carbon-emission calculation to compare siting scenarios under service-utility, travel-efficiency, and lower-carbon priorities. The analysis is intended as a city-specific, mobility-informed scenario evaluation rather than a population-level estimate of healthcare demand. The overall research framework and analytical workflow are summarized in Figure 1. The remainder of the manuscript is organized as follows. Section 2 reviews the literature on hospital choice, healthcare travel behavior, spatial and transport factors, and predictive methods for hospital siting. Section 3 describes the study area, data sources and processing, carbon-emission calculation, and the GCN-dominant ensemble framework. Section 4 presents the healthcare travel characteristics of the respondents, model validation, scenario evaluation, and candidate-location results. Section 5 discusses the principal findings, planning implications, and study limitations. Finally, Section 6 presents the conclusions.

**Figure 1:**
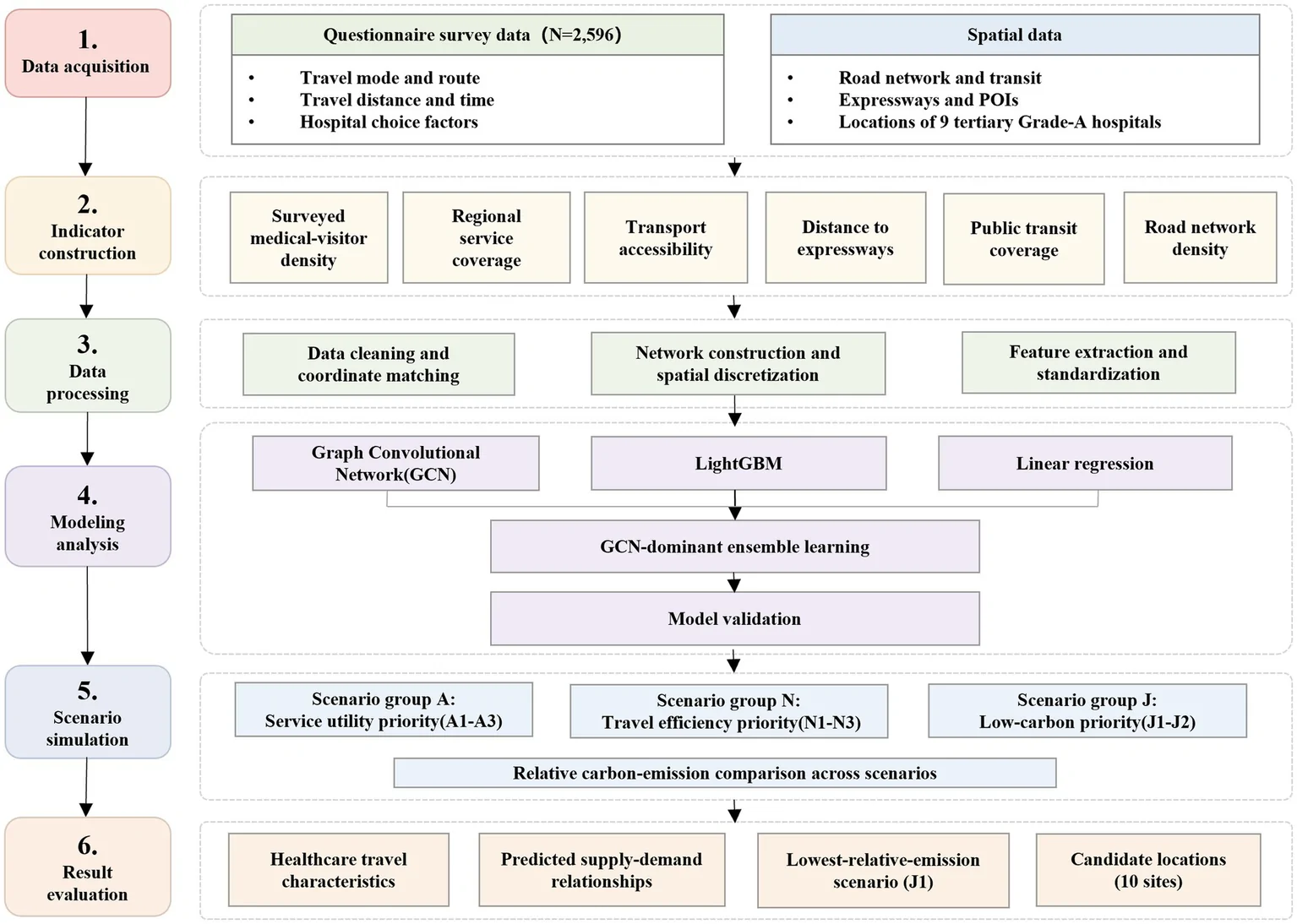
Research framework and methodological workflow of the study. Source: Authors’ own work.

## 2 Literature review

This section reviews previous research on patient hospital choice and healthcare travel behavior, spatial and transport factors relevant to hospital siting, and evaluation and predictive methods used in healthcare planning.

### 2.1 Patient hospital choice and healthcare travel behavior

Hospital choice and healthcare travel behavior reflect interactions among patient needs, provider characteristics, and travel constraints. A scoping review by Victoor et al. showed that patients’ choice of healthcare providers varies across individuals and contexts (20). Empirical studies have documented trade-offs among clinical quality, waiting time, and travel cost. Sivey identified effects of travel and waiting times among English cataract patients (21), while Moscelli et al. showed that distance, quality, and waiting time jointly influenced hospital choice in England (22). Balia et al. found that higher clinical quality attracted some patients to nonlocal hospitals, although distance constrained the range of these choices (23). Survey evidence also reveals substantial heterogeneity. Gao et al. identified distinct patient classes in Shanghai (14), and Kahn et al. found larger relocation-related travel-time effects among public-transport users than among car users (12). US survey studies further link transport barriers with longer access times and delayed care (24, 25). Research in Tianjin associates disability prevalence with medical facilities, bus-route density, road density, and other built-environment factors (26). Together, these findings indicate that transport mode and travel conditions shape realized healthcare access.

Several studies have used observed patient travel to refine healthcare-accessibility assessments. Weinhold et al. derived outpatient catchment standards from acceptable and realized travel distances (11), whereas Liang et al. incorporated multimodal healthcare travel chains into spatial-accessibility measurement (10). Most such studies explain current behavior or evaluate existing access. For prospective hospital siting, observed travel behavior must also be linked to hospital characteristics and the surrounding transport environment.

### 2.2 Spatial and transport factors in hospital siting

The spatial attributes of candidate sites influence whether healthcare services can be used in practice. Accessibility studies commonly examine population demand, resource supply, and spatial impedance, showing how uneven facility distributions produce geographic differences in access (8, 9). Assessments based only on potential opportunities or static supply may overlook actual service use and unmet access needs (9, 27). Candidate sites should therefore be evaluated within the distribution of existing medical resources and local service needs.

Transport conditions determine whether candidate hospital sites can accommodate patient travel needs. Single-mode and static-distance measures can obscure differences in travel time across transport modes, whereas multimodal models better represent accessibility (28). Public-transport access also depends on travel time, cost, walking distance, and transfer convenience (29). Road networks, public-transport services, and multimodal connections are therefore integral to hospital service reach and travel efficiency.

Existing research provides a foundation for hospital-siting assessment by considering medical-resource distribution, spatial accessibility, and the transport environment. Most applications, however, evaluate existing facilities. Prospective siting requires candidate-site conditions to be linked with observed patient travel behavior for supply–demand prediction and scenario evaluation.

### 2.3 Evaluation and predictive methods for hospital siting

Hospital siting and resource allocation have traditionally relied on spatial-accessibility and optimization methods. The two-step floating catchment area method relates medical-resource supply to population demand within a service threshold. Its enhanced form introduces distance decay to avoid treating all locations within a catchment as equally accessible (30, 31). Geographic information systems, multicriteria decision-making, and location-allocation models are also widely used to evaluate candidate sites (7, 32). These methods identify underserved areas but often capture observed patient choice only indirectly.

Discrete-choice models provide another approach to hospital-choice analysis. They commonly use random-utility theory and include distance, waiting time, hospital size, service quality, and patient characteristics (33). Recent work also shows that hospital choice and waiting time can influence one another (34). The patient-hospital relationship is therefore behavioral and dynamic rather than a one-way spatial association.

Combining patient surveys, hospital characteristics, and transport data shifts the task from describing behavior to predicting supply–demand relationships. Linear regression provides an interpretable baseline, while LightGBM can represent high-dimensional and nonlinear feature relationships (35). Patient origins, hospital locations, and observed connections can also be represented as a graph. Graph convolutional and other graph neural networks learn from node attributes and relational structure through message passing (36, 37). These complementary methods motivate an ensemble framework for mobility-informed hospital-siting evaluation.

## 3 Materials and methods

This section describes the study area, data sources and processing, healthcare travel carbon-emission calculation, and the ensemble framework used for supply–demand prediction and scenario evaluation.

### 3.1 Study area

The study focuses on the central urban area of Tianjin. The study area covers 371 km^2^ and has approximately 4.2 million residents. It includes Heping, Hexi, Nankai, Hedong, Hebei, and Hongqiao districts, together with parts of Beichen, Dongli, Jinnan, and Xiqing districts. The area contains concentrated demand for medical services and substantial traffic congestion. It therefore provides a city-specific setting for examining associations among hospital location, healthcare travel, and travel-related carbon emissions.

According to the Notice on Reimbursement for Urban and Rural Residents’ Basic Medical Insurance Outpatient and Emergency Medical Treatment, issued by the Tianjin Municipal Healthcare Security Administration and the Tianjin Municipal Health Commission, Tianjin had 24 tertiary Grade-A hospitals in 2023. Some hospitals operate branch campuses, giving 26 hospital locations in total (Figure 2).

**Figure 2:**
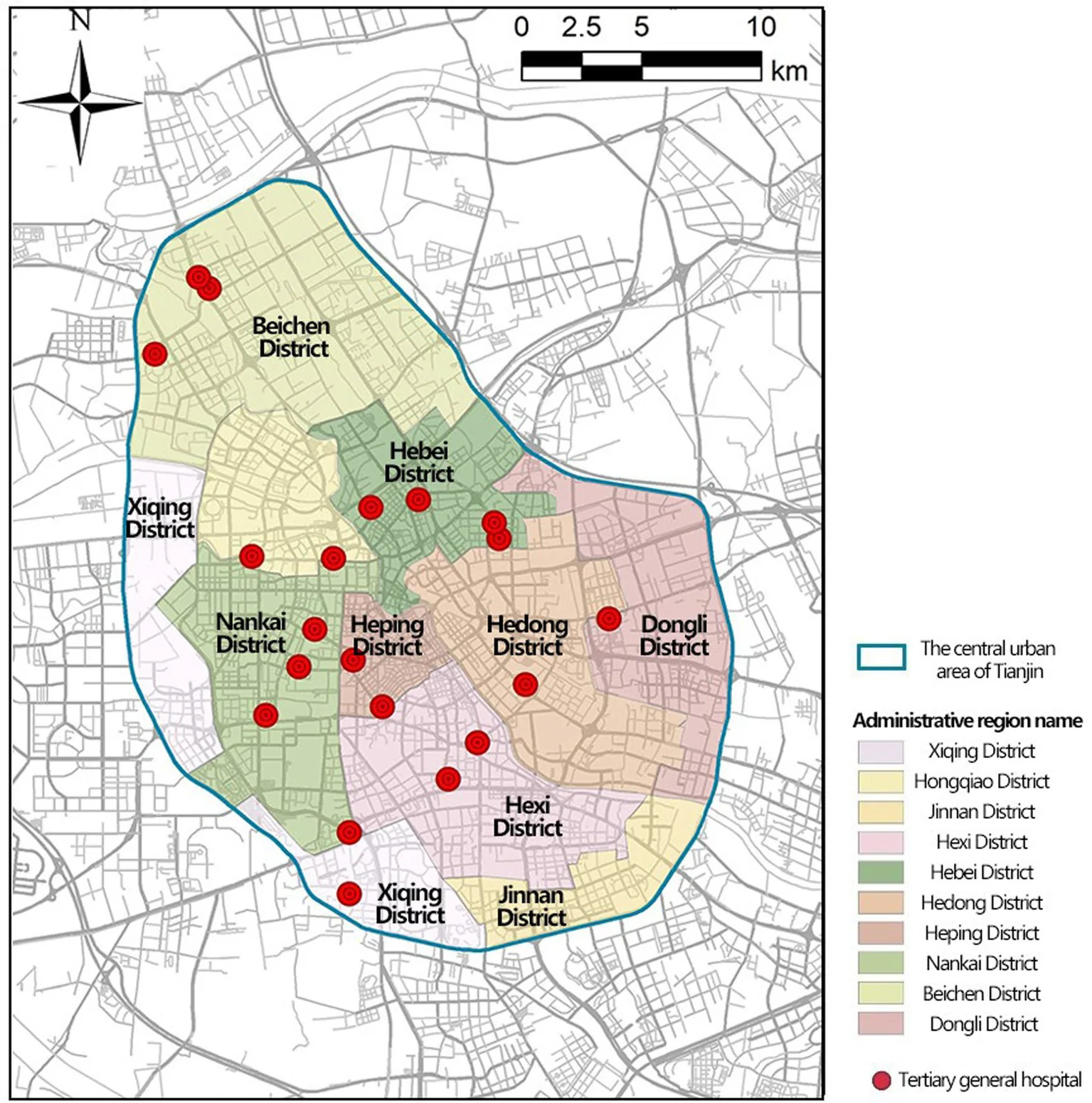
Distribution of tertiary Grade-A hospitals in Tianjin’s central urban area. Source: Authors’ own work.

Study hospitals were selected based on hospital size and local traffic conditions. The Medical and Health Facility Layout Plan of Tianjin (2015–2035, 2019 revision) and the Construction Standard for General Hospitals (Jianbiao 110-2021) identify 800–1,500 beds as a relevant scale for new hospital construction. Because several existing tertiary Grade-A hospitals exceed 1,500 beds, the initial screen included hospital sites with more than 800 beds. Before finalizing the hospital sample, the surrounding operating conditions of the initially screened hospitals were checked for temporary disturbances, such as ongoing hospital reconstruction or changes in nearby road-traffic facilities. Nine hospitals were finally selected for the questionnaire survey and subsequent analysis (Table 1). The analysis is limited to these selected hospitals and does not represent all hospital types or sites in Tianjin.

**Table 1:** Selected tertiary Grade-A hospitals with more than 800 beds in central Tianjin.

Administrative region name	Hospital name	Hospital ID
Hebei district	Joint Logistics Support Force Hospital 983 (Hebei Branch)	H1
	Tianjin Fourth Central Hospital	H2
	Second Affiliated Hospital of Tianjin University of Traditional Chinese Medicine	H3
Nankai district	Tianjin Rehabilitation and Convalescence Center of Joint Logistics Support Force	H4
	Tianjin First Central Hospital	H5
	First Affiliated Hospital of Tianjin University of Traditional Chinese Medicine (North Campus)	H6
	Nankai Hospital of Tianjin Integrative Medicine	H7
Hedong district	Tianjin Third Central Hospital	H8
Hexi district	Tianjin Medical University Second Hospital	H9

### 3.2 Data sources and processing

The study combined hospital-based survey data with multi-source spatial and transport data to construct the behavioral and environmental inputs for the analysis.

#### 3.2.1 Healthcare travel behavior survey

The survey was designed to capture respondents’ residential origins, healthcare travel characteristics, travel intentions, and hospital-choice factors.

A random-intercept questionnaire survey was conducted at hospital entrances, outpatient departments, and inpatient departments of the nine selected hospitals from 15 May to 15 June 2023, between 9:00 and 17:00 each day. At the Tianjin Rehabilitation and Convalescence Center of the Joint Logistics Support Force (H4), access to the inpatient department was restricted; therefore, the inpatient department was not included as a sampling location at this hospital. A total of 3,000 questionnaires were distributed, and 2,596 valid questionnaires were obtained, corresponding to an effective response rate of 86.53%. The numbers of valid questionnaires obtained at H1–H9 were 301, 303, 301, 183, 301, 303, 301, 302, and 301, respectively. Both patients and accompanying persons, including first-time and return visitors, were eligible to participate. Respondent type and visit frequency were recorded in the questionnaire, and each participant completed the questionnaire only once.

An English translation of the complete questionnaire, including all response options and the skip or display rules for conditional questions, is provided in Supplementary material S1. The questionnaire was revised after a small-sample pilot to improve question clarity and response options. It contained 19 questions in four sections (Table 2). Residential areas were identified from regulatory planning index maps published by the Tianjin Municipal Bureau of Planning and Natural Resources. The study included 165 residential areas covering the administrative units within the study area. Cleaned questionnaire records were entered into a database for analysis.

**Table 2:** Types of data collected in the healthcare travel behavior survey.

Data category	Data name	Data description	Data source
Healthcare travel behavior	Respondent attributes	Includes respondents’ residential districts and companions during hospital visits.	Questionnaire survey
	Travel behavior characteristics	Captures visit frequency, transport mode, and travel times.	
	Travel intentions	Includes willingness to use taxi/private car or public transport under specified travel conditions, together with acceptable healthcare travel-distance and travel-time ranges.	
	Hospital choice factors	Identifies key factors influencing hospital selection.	

#### 3.2.2 Multi-source spatial data and indicator extraction

Road networks, bus and metro stations, expressways, and related spatial data were obtained from OpenStreetMap and the Gaode Maps API. ArcGIS 10.2 was used to divide the study area into 200 m × 200 m grid cells. Approximately 9,275 cells were generated for extraction of the six spatial and transport indicators. The resulting high, medium, and low categories were used as indicator settings to construct and compare different scenarios in the subsequent analysis.

1. Surveyed medical-visitor density (X1): To integrate the questionnaire data with the grid-based spatial indicators, the surveyed visitors in each residential area were allocated equally among its 200 m × 200 m grid cells. Density was calculated by dividing the estimated number of surveyed visitors within a 3 km radius of each grid center by the corresponding circular area. Natural breaks classified values as high (14.75–68.78 persons/km^2^), medium (5.18–14.75 persons/km^2^), or low (0–5.18 persons/km^2^).
2. Regional service coverage (X2): A service circle with a radius of 3 km was generated around each tertiary Grade-A hospital. The natural breaks method was used to classify the values into three levels: high (4–5 service circles), medium (2–3 service circles), and low (0–1 service circle).
3. Transport accessibility (X3): Travel speeds were assigned according to road hierarchy, with 80 km/h for expressways, 60 km/h for arterial roads, 40 km/h for secondary arterial roads, and 30 km/h for branch roads. Network Analyst was used to calculate the shortest travel time from each grid center to the nearest hospital. The natural breaks method was used to classify the values into three levels: high (0–25.12 min), medium (25.12–33.62 min), and low (33.62–45.19 min).
4. Distance to expressways (X4): The expressway network in the central urban area of Tianjin was extracted, and the straight-line distance from each grid center to the nearest expressway was calculated. The values were classified into three levels: high (<500 m), medium (500–1,000 m), and low (>1,000 m). A higher level indicates closer proximity to an expressway.
5. Public transit coverage (X5): Using 400 m as the service radius for bus stops and 800 m as the service radius for metro stations, the total number of bus and metro stations covered by each grid was counted. The natural breaks method was used to classify the values into three levels: high (11–58 stops), medium (3–10 stops), and low (0–2 stops).
6. Road network density (X6): Taking each grid center as the center point, a 2 km radius buffer was created. Road network density was calculated by dividing the total road length within the buffer by the buffer area (12.57 km^2^). The natural breaks method was used to classify the values into three levels: high (1.90–3.66 km/km^2^), medium (1.07–1.90 km/km^2^), and low (0–1.07 km/km^2^) (Table 3).

**Table 3:** Summary of spatial and transport indicator levels.

Indicators	High level (1)	Medium level (2)	Low level (3)
Surveyed medical-visitor density (persons/km^2^)	14.75–68.78	5.18–14.75	0–5.18
Regional service coverage (number of facilities)	4–5	2–3	0–1
Transport accessibility (minutes)	0–25.12	25.12–33.62	33.62–45.19
Distance to expressways (meters)	<500	500–1,000	>1,000
Public transit coverage (number of stops)	11–58	3–10	0–2
Road network density (km/km^2^)	1.90–3.66	1.07–1.90	0–1.07

### 3.3 Healthcare travel and carbon emissions calculation

Healthcare travel-related carbon emissions were calculated based on travel distance, travel frequency, travel time, traffic conditions, and the corresponding emission coefficients. The calculation followed the approach reported by Feng et al. (4). The Gaode driving-route API supplied route and traffic-condition information for trips between respondent origins and the selected hospitals. These inputs were used in the study’s carbon-emission calculation, as expressed in the following formula.

∑C=F×L×T×D×K

In this equation, C represents healthcare travel-related carbon emissions (t CO₂); F represents healthcare travel frequency; L represents the total travel distance along the road segments of the driving route (km); T represents the travel time through the corresponding road segments (h); D represents the congestion category of each road segment; and K represents the carbon-emission coefficient corresponding to the congestion category of the road segment [t CO₂/(km·h)]. Traffic conditions were classified as basic free flow, mild congestion, moderate congestion, and severe congestion according to the congestion delay index, and the corresponding emission coefficients are reported in Table 4. Travel modes were recorded as walking/cycling, bus/metro, and taxi/private car. Carbon emissions were estimated only for the combined taxi/private-car category, whereas walking/cycling and bus/metro were treated as low-carbon modes and excluded from the carbon-emission calculation.

**Table 4:** Motor vehicle carbon emissions under different traffic conditions.

Traffic condition	Basic free flow	Mild congestion	Moderate congestion	Severe congestion
CDI range	[1.0, 1.5)	[1.5, 1.8)	[1.8, 2.2)	[2.2, +∞)
Total emissions per 1 km per hour [t·(h·km)^−1^]	0.986	1.576	2.004	3.208
Total emissions per hour per kilometer of urban arterial road [t·(h·km)^−1^]	0.592	0.946	1.202	1.925

### 3.4 GCN-dominant ensemble framework for supply–demand prediction and carbon-emission scenario evaluation

This subsection describes the construction, integration, validation, and application of the GCN-dominant ensemble framework.

This study uses a GCN-dominant ensemble framework to predict respondent-hospital supply–demand relationships and compare relative healthcare travel-related carbon-emission levels. The framework combines questionnaire data, hospital spatial features, the surrounding road-traffic environment, and urban congestion information. GCN, LightGBM, and linear-regression components generate complementary predictions, which are then integrated for carbon-emission calculation and scenario evaluation.

#### 3.4.1 Feature data input and graph structure construction

The input module organizes questionnaire responses, hospital spatial features, surrounding road-traffic conditions, and urban congestion data. Survey responses provide healthcare travel behavior and preference information, while spatial measurement and the Gaode Maps API provide hospital, road, and congestion attributes. After preprocessing and normalization, hospital sites and respondent-origin demand are represented as graph nodes. Hospital spatial information and respondent healthcare-demand and preference information are assigned as node attributes, while edges represent respondent–hospital supply–demand relationships. Euclidean distances for all respondent–hospital pairs are converted using a distance threshold into a binary relationship matrix (1 = matched; 0 = unmatched), which is used to construct the GCN adjacency matrix. Self-loops and symmetric degree normalization are applied during graph convolution. The hospital selected by each respondent serves as the supervised label, and the model outputs the predicted probability of selecting each hospital.

#### 3.4.2 Identification and Learning of Supply–Demand Relationship

The GCN component uses the graph representation to learn associations among respondent-origin demand, hospital spatial features, and the surrounding transport environment. The supervised task predicts the strength of respondent–hospital relationships under the spatial conditions represented in the study data.

#### 3.4.3 Multi-model ensemble integration

The framework combines GCN, LightGBM, and linear-regression components to improve prediction stability. Each component is trained separately and generates hospital-choice scores for the test data. Five candidate models—random forest, a conventional deep-learning model, linear regression, LightGBM, and GCN—were compared in terms of accuracy, efficiency, interpretability, adaptability, and robustness. Based on this comprehensive assessment, GCN, LightGBM, and linear regression were selected and integrated through soft voting, with weights of 70, 15, and 15%, respectively. The resulting ensemble prediction captures respondents’ predicted hospital choices and is subsequently used for scenario evaluation.

#### 3.4.4 Data propagation and carbon emission transformation

The data-propagation module combines observed respondent–hospital relationships, predicted relationships, and urban congestion data from the Gaode Maps API. The healthcare travel carbon-emission calculation is then applied to convert predicted relationships into relative emission results for the evaluated spatial-configuration scenarios. This step links supply–demand prediction to the subsequent lower-carbon scenario comparison.

#### 3.4.5 Model validation

Model validation comprised sampling, simulation, comparison, and accuracy assessment. Cluster sampling replaced one observed hospital with a candidate location selected according to spatial characteristics. Random sampling then generated more than 2,000 candidate locations for prediction and scenario evaluation. The replacement samples were used for internal validation, while randomly selected locations were used for candidate-site prediction. Model-generated relationships were compared with observed respondent-hospital relationships to calculate the reported prediction accuracy.

#### 3.4.6 Multi-condition scenario simulation and scenario evaluation

The framework compared eight scenarios organized into three objective-based groups: service utility (A1–A3), travel efficiency (N1–N3), and low-carbon effect priority (J1–J2). The groups represented service-utility, travel-efficiency, and lower-carbon priorities. Each indicator was classified as high, medium, or low, and the resulting combinations were compared using the study’s relative carbon-emission categories.

In the service-utility group, surveyed medical-visitor density and regional service coverage were varied while transport indicators were held constant. In the travel-efficiency group, transport accessibility, distance to the nearest expressway, public transit coverage, and road network density were varied while the service-space indicators were held constant. The lower-carbon group compared the relative carbon-emission levels across the evaluated combinations and selected the lowest category (Table 5).

**Table 5:** Scenario design under different simulation objectives.

Simulation objective	Dependent variable	Independent variables	Control variables
Service utility priority	Y1	X1 + X2	X3 + X4 + X5 + X6 + b
Travel efficiency priority	Y2	X3 + X4 + X5 + X6	X1 + X2 + b
Low-carbon effect priority	Y3	Y3 is derived by comparing Y1 or Y2 under different objective scenarios; the minimum Y3 value is selected.	

## 4 Results

This section presents respondents’ healthcare travel characteristics, internal validation results, comparisons across planning scenarios, and the resulting candidate locations.

### 4.1 Healthcare travel characteristics among survey respondents

This subsection summarizes respondents’ hospital choices and travel modes, together with their reported travel-distance and travel-time tolerance.

#### 4.1.1 Spatial choice and travel modes for medical visits

Residential-zone matching showed that the three selected hospitals in Hebei District served respondents from Hebei, Hedong, Dongli, and Beichen districts. The three selected hospitals in Nankai District also served respondents from Heping, Hedong, Hongqiao, and Xiqing districts. The selected hospitals in Hedong and Hexi districts attracted some respondents from Dongli and Jinnan districts, respectively (Figure 3). Within this hospital-based sample, respondents most often visited hospitals in their residential district or a neighboring district.

**Figure 3:**
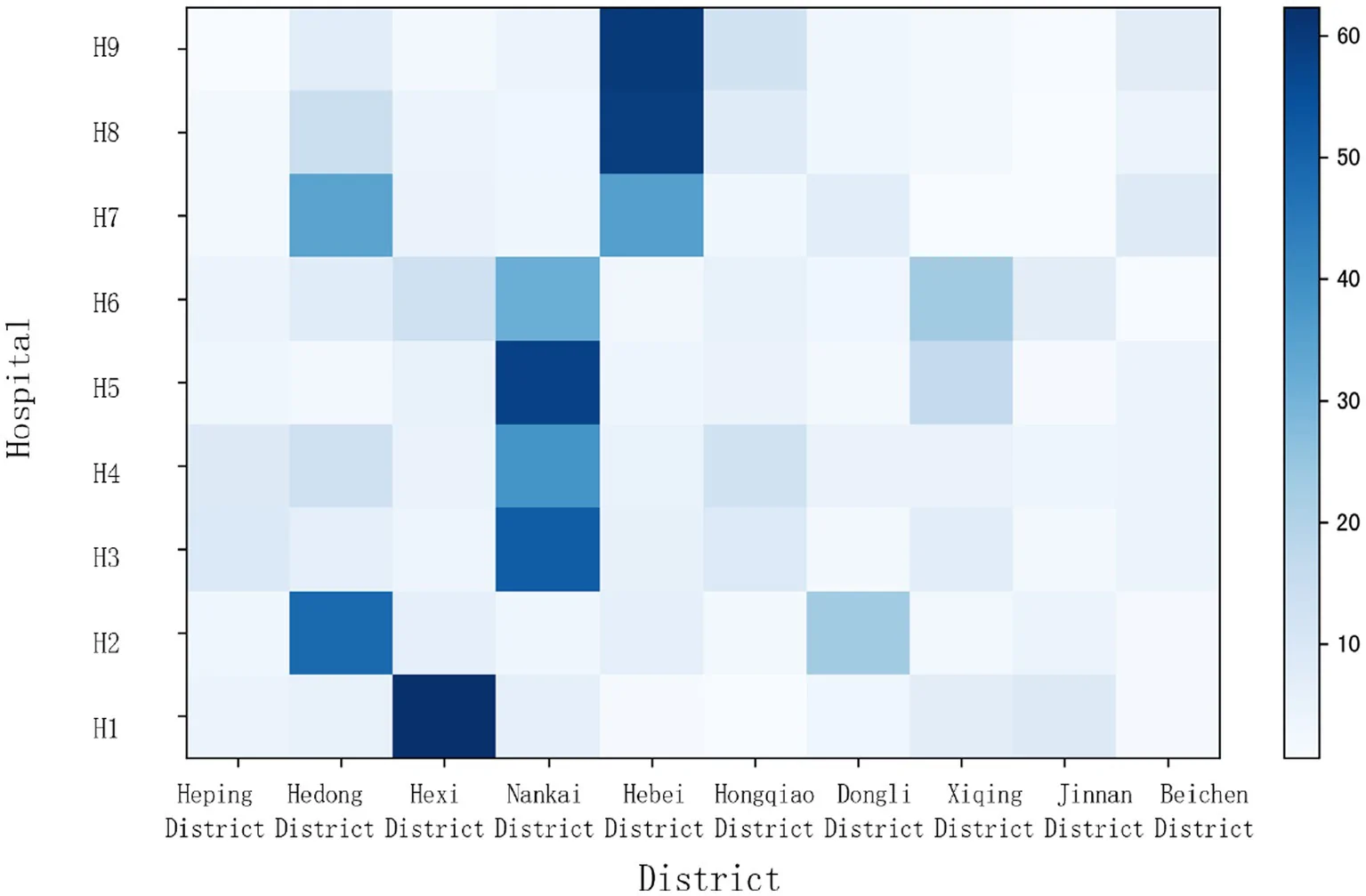
Distribution of residential administrative districts among respondents visiting the nine tertiary Grade-A hospitals. Source: Authors’ own work.

The main reasons for hospital choice were grouped into three categories: proximity and convenient transport, overall reputation and public recognition, and specialty-specific medical capacity (Figure 4). Specialty-specific services accounted for the largest share, followed by overall reputation and proximity. Across the nine hospitals, 39.74–64.45% of respondents selected specialty capacity as their main reason. Reputation accounted for 15.30–37.21%, while proximity and transport convenience accounted for 11.55–32.12%.

**Figure 4:**
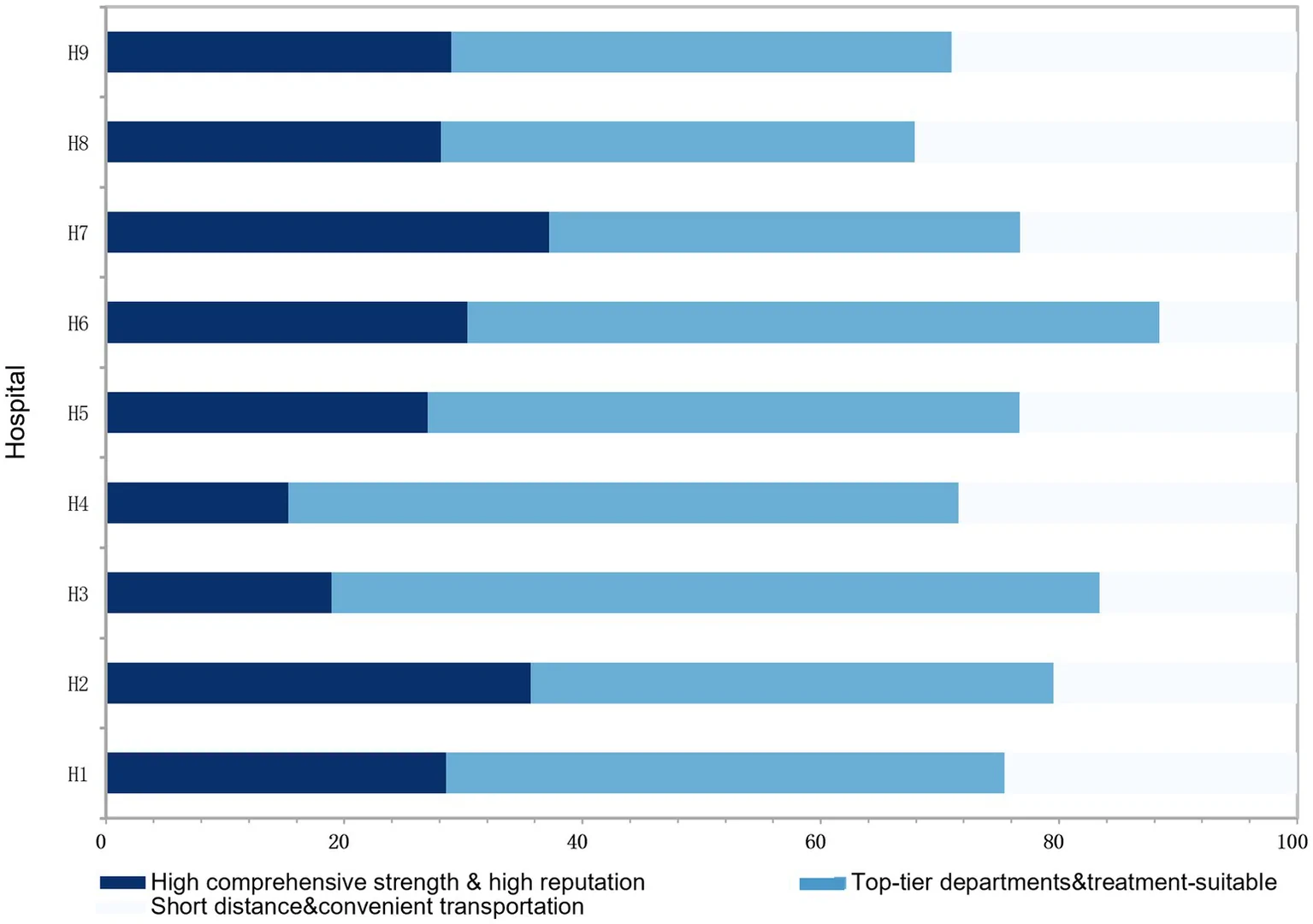
Main reasons for respondents’ hospital choice. Source: Authors’ own work.

Travel modes differed across the nine hospitals when questionnaire responses were considered alongside local transport indicators (Figure 5). Private car or taxi was the most common mode, accounting for 31.02–57.92% of visits. Walking or cycling accounted for 20.86–30.56%, bus travel for 12.96–22.92%, and metro travel for 3.83–22.44%.

**Figure 5:**
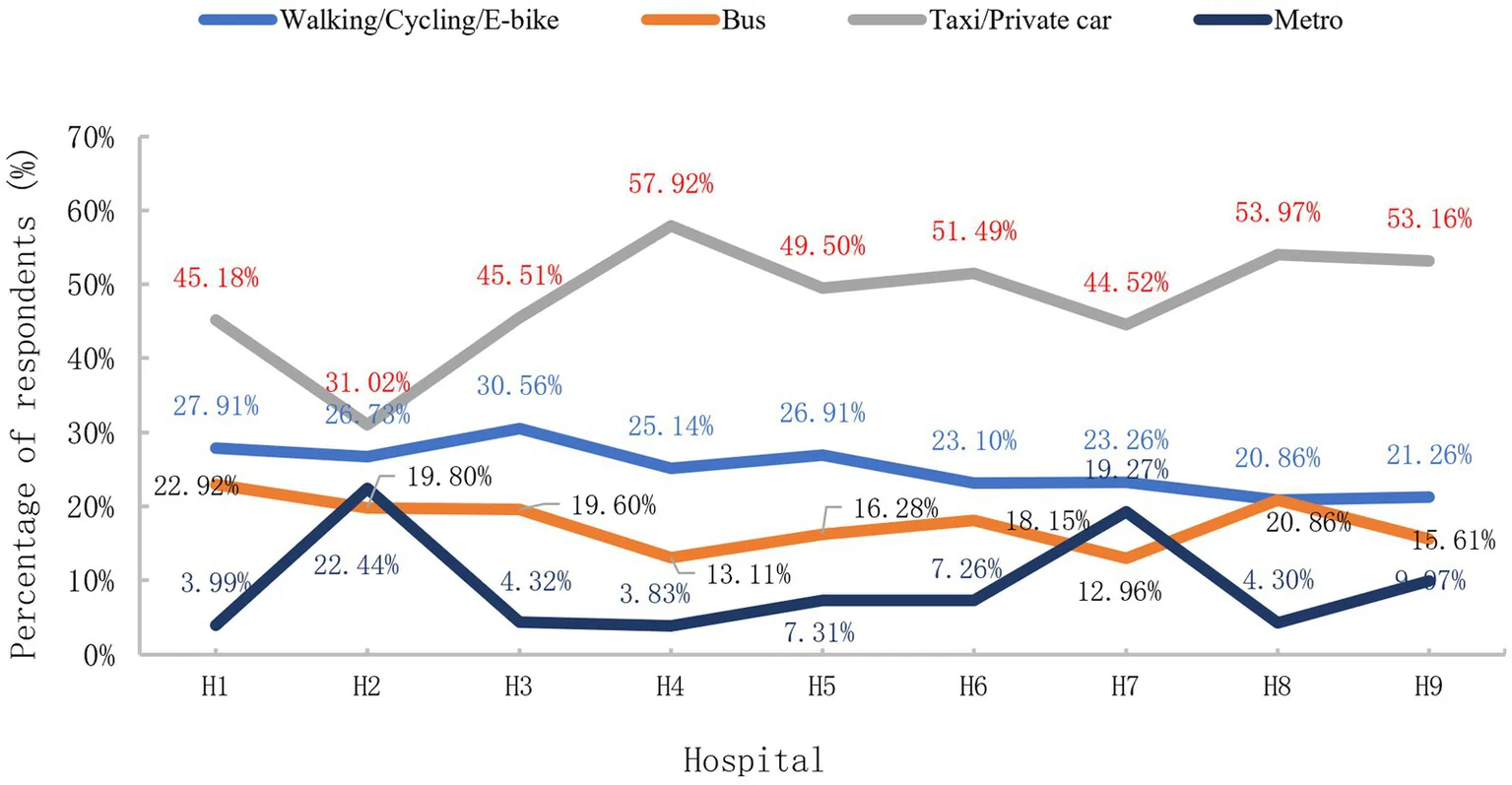
Variation in travel modes among survey respondents. Source: Authors’ own work.

Responses regarding the conditions influencing taxi/private car use were examined separately (Figure 6). Reported conditions included inconvenient public transport, disease severity, faster access from expressway proximity, and long travel distance or time. Inconvenient public transport accounted for 16.23–32.01% of all hospital visitors, while disease severity accounted for 8.74–18.94%. Expressway proximity and faster access accounted for 5.61–16.17%, whereas long distance and travel time accounted for 0.66–6.60%. These descriptive results identify public-transport convenience and travel efficiency as relevant factors in lower-carbon healthcare travel.

**Figure 6:**
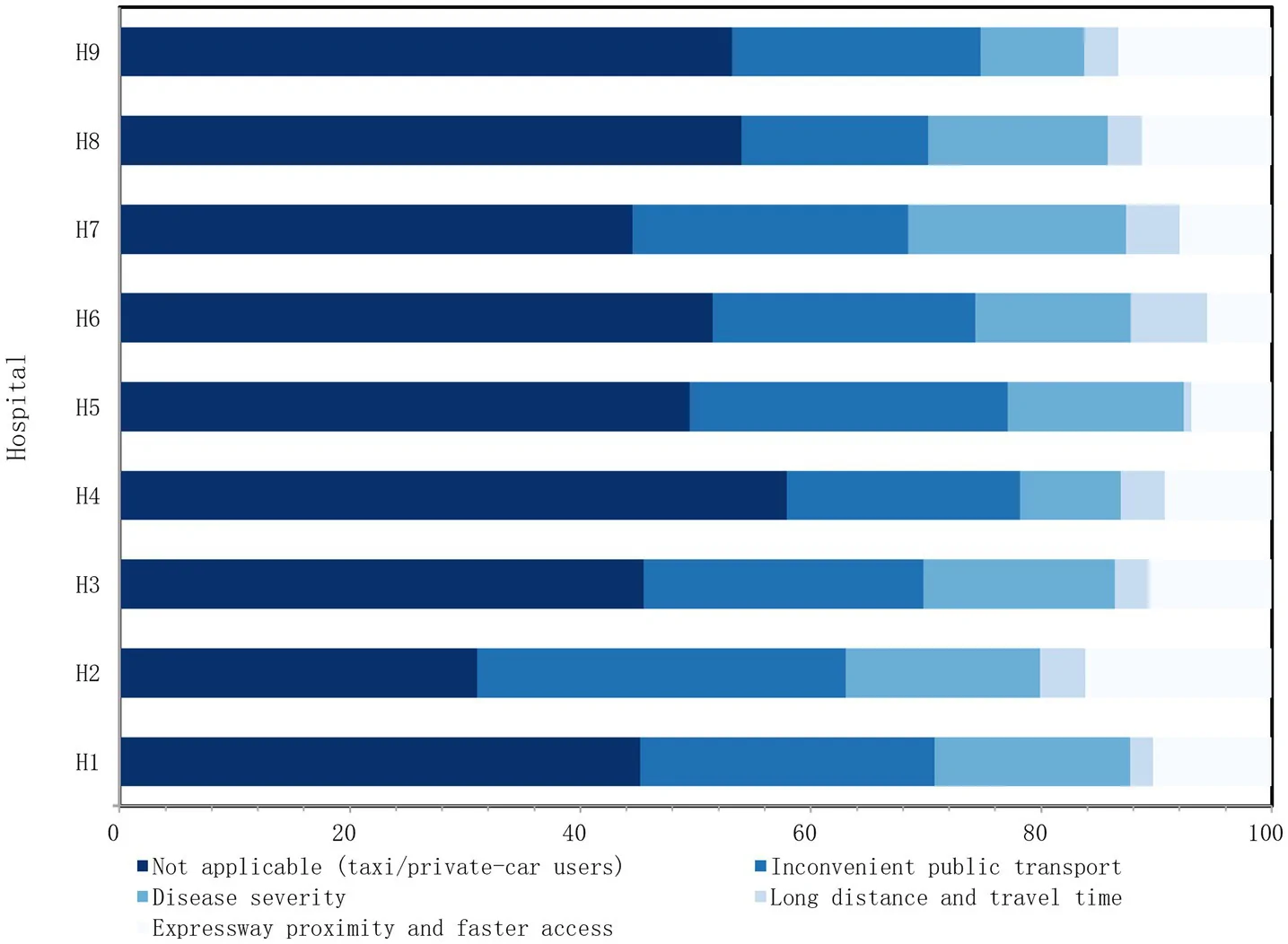
Conditions influencing potential taxi-private car use among respondents traveling to and from tertiary Grade-A hospitals. Source: Authors’ own work.

#### 4.1.2 Travel-distance and travel-time tolerance

Reported travel-distance tolerance was grouped into five categories: 0–3 km, 3–5 km, 5–10 km, 10–20 km, and more than 20 km. Across the nine hospitals, the corresponding proportions ranged from 1.00 to 15.95%, 8.91 to 42.19%, 31.56 to 50.27%, 10.30 to 33.00%, and 0.66 to 10.23%, respectively (Figure 7).

**Figure 7:**
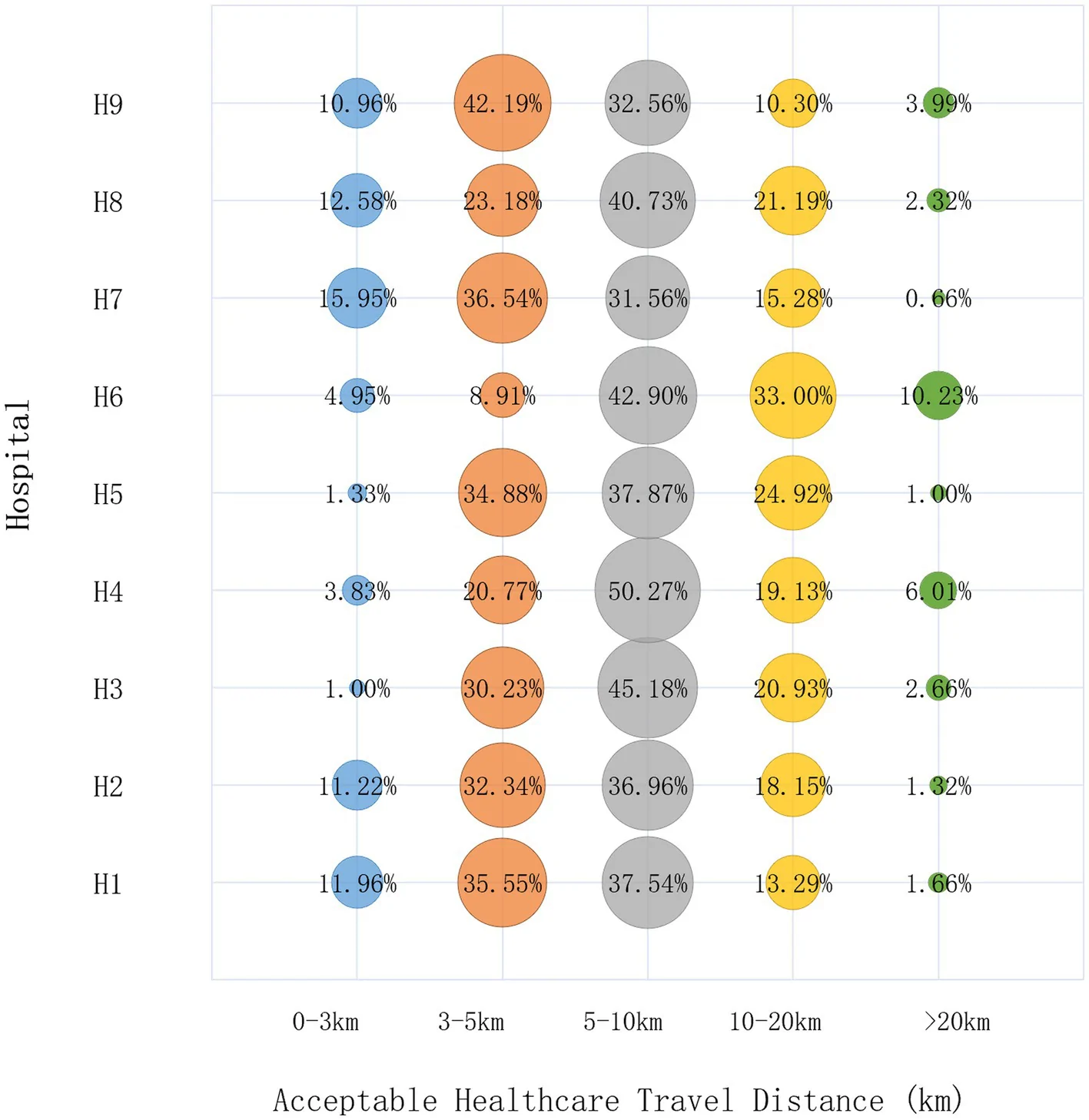
Respondents’ reported travel-distance tolerance. Source: Authors’ own work.

Reported travel-time tolerance was grouped into 0–15, 15–30, 30–60, 60–120, and more than 120 min. Across the nine hospitals, the corresponding proportions ranged from 4.62 to 15.28%, 35.55 to 52.49%, 32.56 to 48.63%, 0.55 to 11.22%, and 0.00 to 1.33%, respectively (Figure 8). Within the surveyed sample, most respondents reported travel tolerances within 20 km and 60 min.

**Figure 8:**
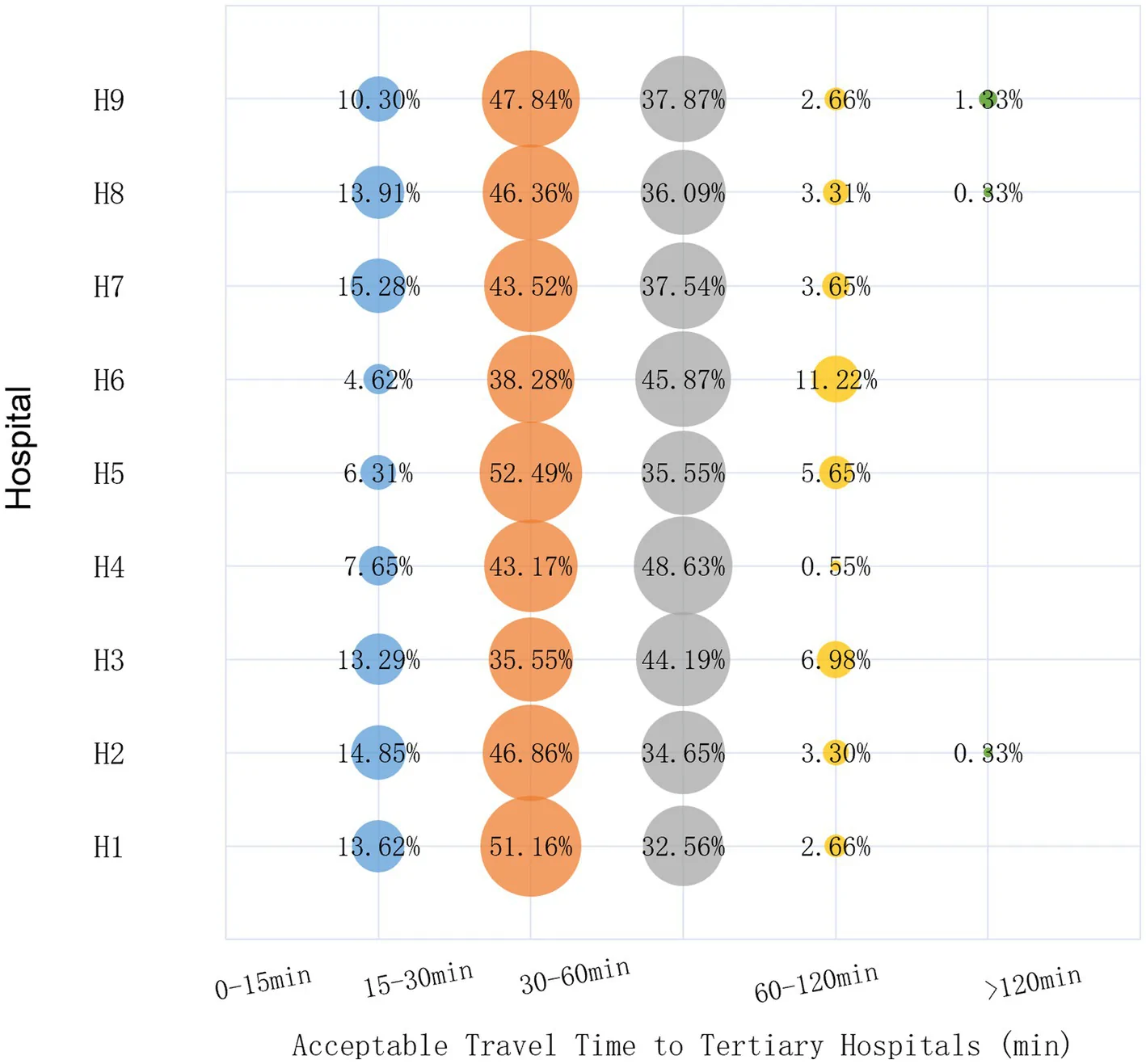
Respondents’ reported travel-time tolerance. Source: Authors’ own work.

Respondents visiting the First Affiliated Hospital of Tianjin University of Traditional Chinese Medicine (North Campus) reported relatively high tolerance for both distance and time. A similar pattern was observed at the Second Affiliated Hospital of Tianjin University of Traditional Chinese Medicine. These patterns are consistent with respondents’ emphasis on specialty services. By contrast, respondents who prioritized proximity and transport convenience generally reported lower travel tolerance.

When direct public transport was available, willingness to shift from higher-carbon modes varied with the number of route stops. Across the nine hospitals, willingness ranged from 8.64 to 18.21% for no more than 5 stops, 8.25 to 22.92% for 6–10 stops, 6.60 to 19.13% for 11–15 stops, and 1.66 to 4.65% for more than 15 stops. The proportion unwilling to use public transport ranged from 3.99 to 28.24% (Figure 9). Thus, reported willingness declined as the number of stops and associated travel burden increased.

**Figure 9:**
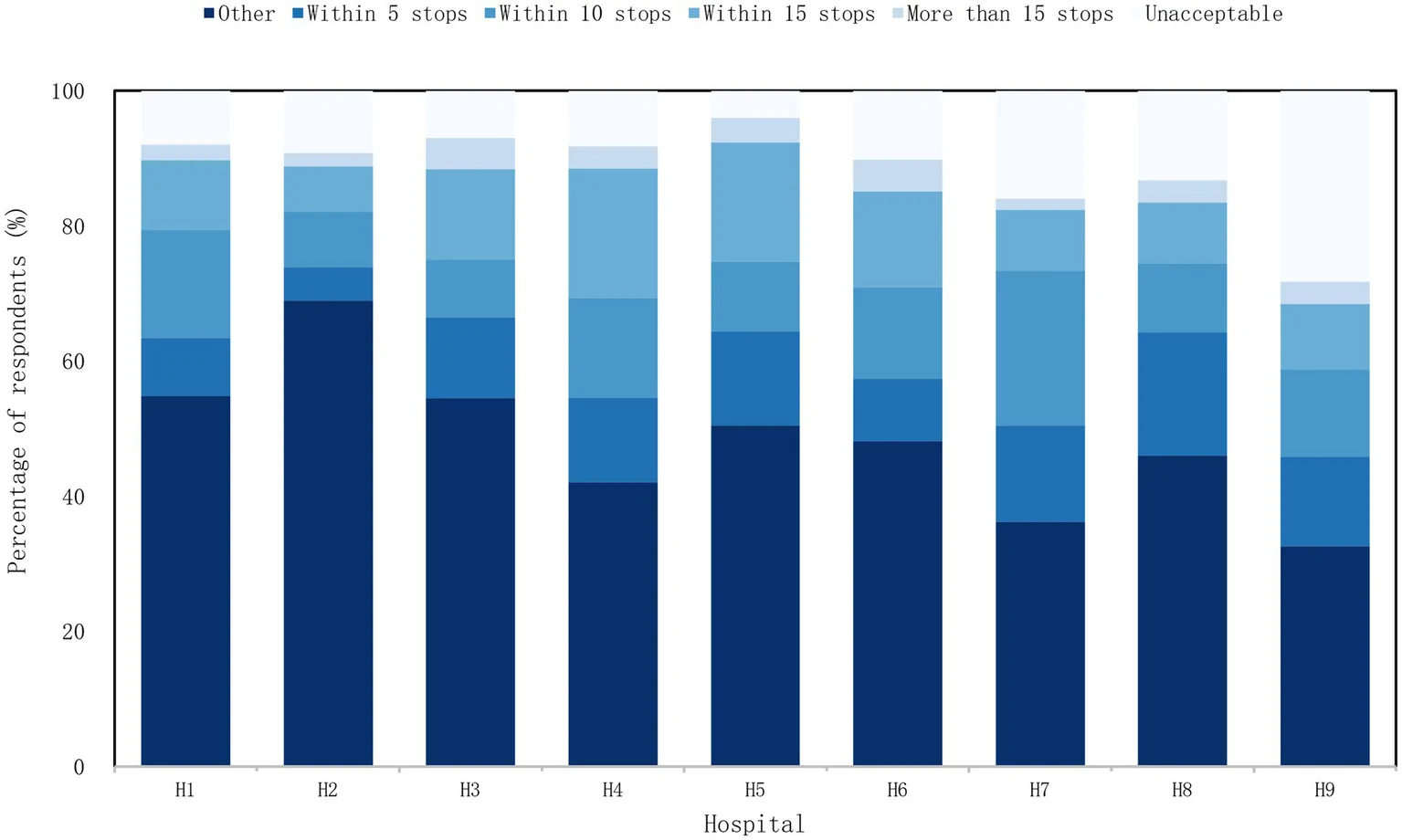
Respondents’ willingness to use direct public transport for hospital visits. Source: Authors’ own work.

### 4.2 Internal validation of the ensemble prediction framework

The internal validation compared model-generated and observed respondent-hospital relationships. Prediction accuracy remained above 90% under this procedure. This result indicates that the ensemble captured patterns in the study dataset, although external predictive performance was not assessed.

The internally validated framework was then used to compare candidate locations. It linked respondent travel patterns with hospital spatial characteristics and transport conditions, providing inputs for supply–demand prediction and relative carbon-emission scenario evaluation.

### 4.3 Scenario evaluation under different planning priorities

Eight representative siting scenarios were compared. A1–A3 represented service-utility priorities, N1–N3 represented travel-efficiency priorities, and J1–J2 represented lower-carbon comparisons. The analysis compared their spatial-feature combinations and reported relative carbon-emission levels (Table 6).

**Table 6:** Comparison of spatial-feature combinations and relative carbon-emission levels across scenarios.

Scenario ID	Objective priority	Parameter combination (X1, X2, X3, X4, X5, X6)	Relative carbon emission level
A1	Service utility priority	(1, 2, 1, 1, 1, 2)	Low
A2	Service utility priority	(2, 2, 1, 1, 1, 2)	High
A3	Service utility priority	(3, 2, 1, 1, 1, 2)	Medium
N1	Travel efficiency priority	(2, 2, 2, 1, 1, 1)	Low
N2	Travel efficiency priority	(2, 2, 1, 1, 1, 1)	Medium
N3	Travel efficiency priority	(2, 2, 2, 2, 2, 2)	High
J1	Low-carbon effect priority	(2, 1, 1, 3, 1, 2)	Lowest
J2	Low-carbon effect priority	(3, 2, 3, 1, 2, 2)	High

#### 4.3.1 Service utility priority scenario

A1–A3 compared high, medium, and low surveyed medical-visitor density while holding X3–X6 constant. A1 (X1 = 1) had a low relative carbon-emission level, A2 (X1 = 2) had a high level, and A3 (X1 = 3) had a medium level. Among these three evaluated scenarios, A1 had the lowest reported category.

#### 4.3.2 Travel efficiency priority scenario

N1–N3 compared three transport-environment configurations while holding the service-space indicators constant (X1 = 2, X2 = 2). N1 (X3 = 2, X4 = 1, X5 = 1, X6 = 1) had a low relative carbon-emission level. N2 (X3 = 1, X4 = 1, X5 = 1, X6 = 1) had a medium level, and N3 (X3 = 2, X4 = 2, X5 = 2, X6 = 2) had a high level. N1 therefore had the lowest reported category among these scenarios.

#### 4.3.3 Low-carbon effect priority scenario

The lower-carbon comparison identified J1 (2, 1, 1, 3, 1, 2) as the scenario with the lowest relative carbon-emission level.J2 (3, 2, 3, 1, 2, 2), characterized by medium regional service coverage and medium public transit coverage, had a high relative carbon-emission level. J1 was classified as the lowest of the evaluated scenarios (Figure 10).

**Figure 10:**
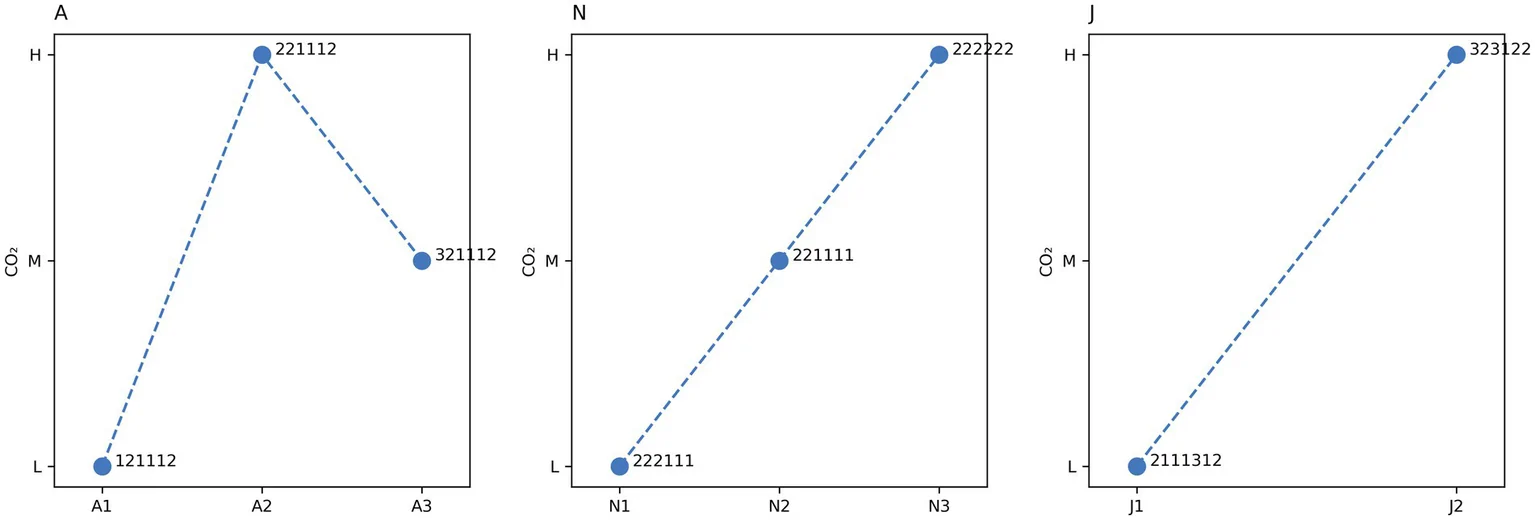
Simulation results of scenarios A, N, and J. Source: Authors’ own work.

### 4.4 Candidate locations under the lowest-relative-emission scenario

Within the evaluated scenario set, J1 was associated with the lowest relative carbon-emission level. Its indicator ranges were 5.18–14.75 surveyed medical visitors/km^2^, 4–5 facilities for regional service coverage, 0–25.12 min for transport accessibility, more than 1,000 m from expressways, 11–58 public transit stops, and 1.07–1.90 km/km^2^ for road network density. The model was then used to identify the ten locations with the lowest predicted relative carbon-emission levels from the J1 candidate-location set (Figure 11).

**Figure 11:**
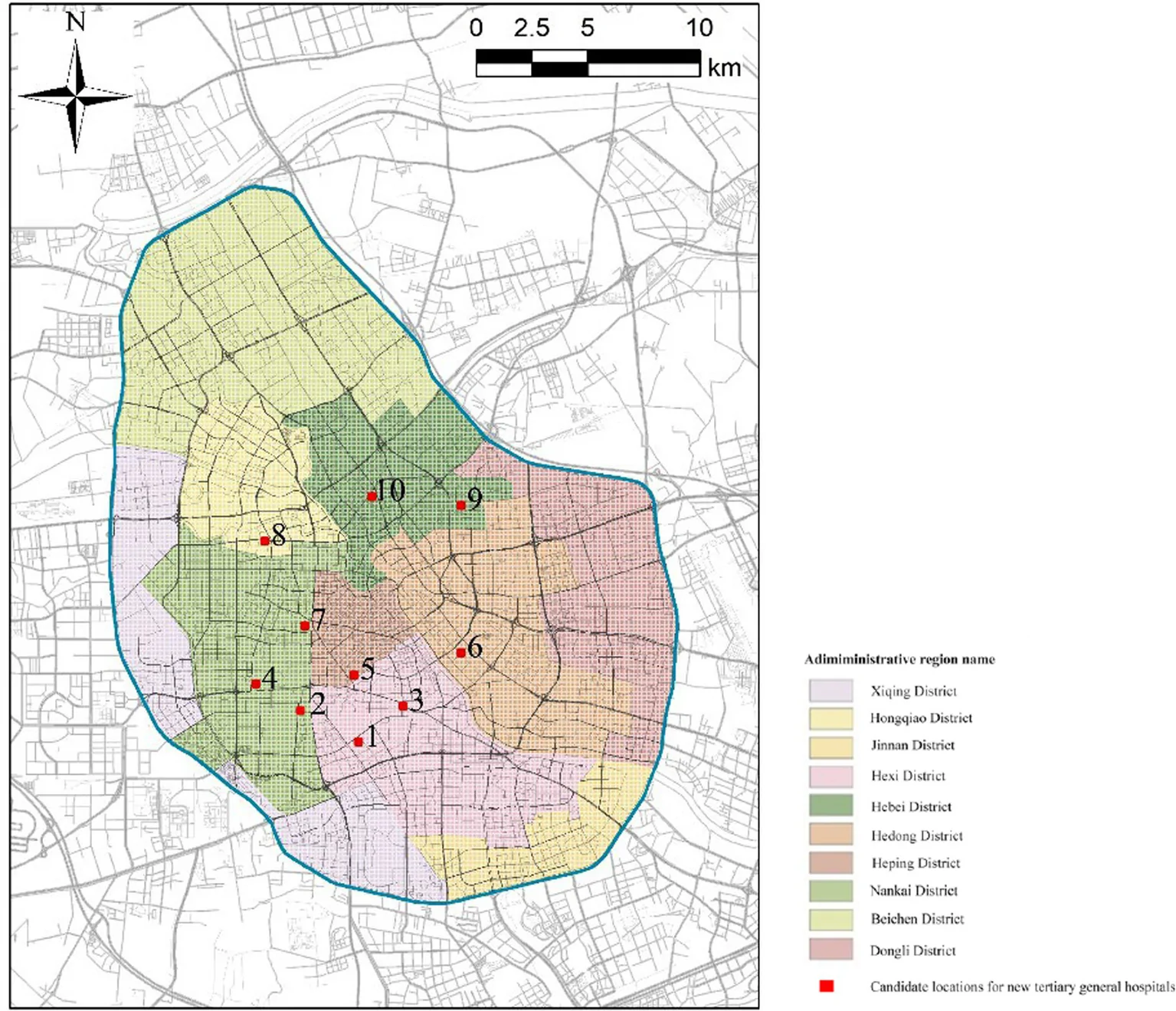
Site locations for newly built tertiary Grade-A hospitals in central Tianjin. Source: Authors’ own work.

## 5 Discussion

This section interprets the healthcare travel findings, transport implications, scenario-evaluation results, planning relevance, and study limitations.

### 5.1 Hospital choice and reported travel tolerance

Within the surveyed sample, travel-distance and travel-time tolerance varied with respondents’ reported reasons for hospital choice. Those who emphasized specialty services, hospital reputation, or treatment suitability generally accepted longer trips. Respondents who prioritized proximity and transport convenience reported lower tolerance for time and distance.

These findings are consistent with hospital choice as a trade-off among service quality, waiting costs, travel costs, and medical needs. Lippi Bruni et al. linked hospital choice to changes in waiting time and clinical quality (38). Monfared et al. also identified distance, health status, perceived quality, and referral relationships as relevant factors (39). Service radius or straight-line distance alone therefore cannot represent the travel patterns observed in this sample. Hospital-siting evaluations should consider stated choice reasons and reported travel tolerance alongside spatial coverage.

### 5.2 Healthcare travel mode choice and transport conditions

Private-car and taxi use was associated with reported public-transport inconvenience, disease severity, expressway proximity, and travel distance or time. When direct public transport was available, some respondents reported willingness to shift to public transport. That willingness decreased as route stops and travel burden increased.

Public-transport accessibility depends on route directness, transfer convenience, and total travel time, not only on stop coverage. Gan et al. found that public transport can improve equity in access to high-level healthcare (40). Boisjoly et al. likewise showed that social and spatial factors constrain hospital access in Canadian metropolitan regions (41). For the surveyed Tianjin hospitals, public-transport connections should therefore be considered alongside road access. This approach may reduce travel burdens for transit-dependent and mobility-limited patients while supporting lower-carbon travel.

### 5.3 Hospital-siting scenario evaluation using predicted supply–demand relationships

The scenario evaluation combined respondent travel behavior, hospital spatial characteristics, and transport-environment indicators. The lowest relative carbon-emission levels varied across the service-utility, travel-efficiency, and lower-carbon scenario groups. Hospital siting should therefore be assessed as a trade-off among service coverage, travel conditions, and lower-carbon performance rather than as a single coverage problem.

Previous studies have emphasized spatial accessibility, transport conditions, and service equity in hospital planning. Balsa-Barreiro et al. incorporated road characteristics and travel time into a spatial-network assessment (42). Yang et al. compared accessibility before and after new hospital construction (43), while Zhang et al. evaluated the equity effects of reallocating higher-level hospital beds (44). Because accessibility also varies by time and travel mode (45), the present scenario evaluation includes transport accessibility, public transit coverage, road network density, and distance to expressways.

Healthcare travel can also generate substantial greenhouse gas emissions (46). In Tianjin, Dong et al. showed that reallocating pediatric medical facilities can reduce travel-related CO₂ emissions (47). Sánchez-Balseca et al. further demonstrated that travel time, traffic conditions, and vehicle type influence transport-related CO₂ emissions (48). Hospital location may therefore affect both healthcare access and travel-related emissions through changes in distance, time, mode, and traffic conditions.

### 5.4 Implications of multi-objective scenario evaluation for hospital siting

Among the evaluated scenario groups, A1 had the lowest category for the service-utility comparison, N1 had the lowest for the travel-efficiency comparison, and J1 had the lowest overall relative carbon-emission level. J1 combined medium surveyed medical-visitor density, high regional service coverage, high transport accessibility, greater distance from expressways, high public transit coverage, and medium road network density. These findings describe the evaluated combinations and should not be interpreted as a universal optimum beyond the study setting.

The J1 pattern indicates that lower relative carbon-emission performance in this scenario set was associated with a combination of service coverage, transport accessibility, public transport, expressway distance, and road network density. No single indicator determined the resulting category. Mobility-informed hospital siting can therefore complement static facility-coverage assessment by considering travel burden and the feasibility of lower-carbon transport.

### 5.5 Research limitations

This study has several limitations. First, the survey was conducted among visitors to nine selected hospitals in central Tianjin. It does not represent residents who did not seek care, used other facilities, or had unmet healthcare needs. Second, the equal-allocation approach used to derive X1 may smooth spatial heterogeneity within residential areas, and the Jenks natural-break thresholds are specific to the present study area. Future studies should assess the robustness of these methodological choices using alternative spatial-allocation and classification methods. Third, age, income, disease type, and physical mobility were not collected in the questionnaire; therefore, subgroup differences in these characteristics could not be assessed. Fourth, the ensemble was evaluated internally, and its transferability to other cities or hospital systems remains untested. Finally, the carbon-emission assessment uses categorized traffic conditions and reported coefficients. More detailed trajectory, vehicle, and public-transport operating data are needed for precise emission estimates.

## 6 Conclusion

Using 2,596 hospital-based survey responses and spatial-transport data from central Tianjin, this study developed a GCN-dominant ensemble framework for mobility-informed hospital-siting scenario evaluation. Respondents’ reported travel tolerance was concentrated within 20 km and 60 min, while willingness to use direct public transport declined as the number of route stops increased. Among the evaluated scenarios, J1 combined medium surveyed medical-visitor density, high regional service coverage and transport accessibility, greater distance from expressways, high public transit coverage, and medium road network density. J1 had the lowest relative carbon-emission level.

These findings support the inclusion of observed healthcare travel behavior in hospital-siting assessment rather than reliance on static service areas alone. However, the results are specific to surveyed visitors at nine selected hospitals, the study’s indicator definitions, its internal validation procedure, and its relative emission categories. Population-based demand data and external validation are required before the framework can be applied to other cities or used for definitive siting decisions.

## Data Availability

The raw data supporting the conclusions of this article will be made available by the authors, without undue reservation.
