# Supplementary material for: Mobility-informed hospital siting for accessible and lower-carbon healthcare travel: an ensemble learning study in Tianjin, China
Source: Front Public Health. 2026 Sep 14;14:1934861. doi: 10.3389/fpubh.2026.1934861 (PMC13616939; doi:10.3389/fpubh.2026.1934861)
Supplement: Supplementary file 1 [file Table_1.DOCX]

# Appendix

## Appendix A Questionnaire Survey of Tertiary Grade-A General Hospitals in Central Tianjin

1. Hospital visited ( )
（1） Joint Logistics Support Force Hospital 983 (Hebei Branch)
（2） Tianjin Fourth Central Hospital
（3） Second Affiliated Hospital of Tianjin University of Traditional Chinese Medicine
（4） Tianjin Rehabilitation and Convalescence Center of Joint Logistics Support Force (formerly Hospital 464)
（5） Tianjin First Central Hospital
（6） First Affiliated Hospital of Tianjin University of Traditional Chinese Medicine (North Campus)
（7） Nankai Hospital of Tianjin Integrative Medicine
（8） Tianjin Third Central Hospital
（9） Tianjin Medical University Second Hospital
2. Did you visit the outpatient department or the inpatient department? ( )

(1) Outpatient department	(2) Inpatient department

3. In which district of Tianjin do you reside? ( )

(1) Heping District (2) Hexi District (3) Nankai District (4) Hedong District (5) Hebei District

(6) Hongqiao District (7) Beichen District (8) Dongli District (9) Jinnan District (10) Xiqing District

4. Which residential area do you live in? ( )

| Administrative district | Area name and map |
| --- | --- |
| (1) Heping District | 1) Area around Nanshi Park, Poly Plaza, and Tianjin No. 2 Nankai High School 2) Area around Tianjin Eye Hospital and the Institute of Hematology & Blood Diseases Hospital, Chinese Academy of Medical Sciences 3) Area around Songyue Xili residential community, Lebin Department Store, and Youyi Xintiandi Plaza 4) Area around Tianjin Medical University General Hospital and St. Joseph Cathedral (Xikai Church) 5) Area around Tianjin Experimental Primary School and Century Metropolis 6) Area around Tianjin Yaohua High School and Shuangling High School 7) Area around Tianjin Radio and Television Station, Tianjin Medical University, and Tianjin No. 55, No. 90, and No. 21 High Schools 8) Historic neighborhoods including the Five Great Avenues and Central Park 9) Area around Tianjin Concert Hall and Hisense Plaza    |
| (2) Hexi District | 1) Area around Tianjin No. 41 High School and Tianjin Foreign Studies University 2) Area around Tianjin International Trade Shopping Center and People's Park 3) Area around Hexi District Rehabilitation Hospital, Tianjin Haihe High School, and Jiefang South Garden 4) Area around China Overseas Bali Terrace, Tianjin Experimental High School, and Tianjin Hotel 5) Area around Tianjin Children's Hospital 6) Area around Zhongfu Building, Endeli Primary School, and Binhai Hospital 7) Area around Sunshine New Life Plaza and Tianjin No. 42 High School 8) Area around Yile Tiandi Shopping Center and Hexi Hospital 9) Area around Tianta Lake, Huaxia Future Children's Park, and Tianjin Medical University Cancer Institute and Hospital 10) Area around Xinanlou Park, Tianjin MixC, and Tianjin Cultural Center 11) Area around Tianjin Medical University Second Hospital, Hongguang Park, and Tianjin No. 4 High School 12) Area around CapitaMall, Tianjin Bay Park, and Chentangzhuang Station (Metro Line 1) 13) Area around Tianjin University of Sport, Nanhu Primary School, and Tibei Luneng Mansion 14) Area around Tianjin Municipal People's Government, New Century City, and Hexi District Central Primary School (West Campus) 15) Area around Xinye Plaza and Red Star Macalline 16) Area around Fuxing Primary School, Greenway Park, Dongting Park, and Zhujiang Decoration City 17) Area around Mingdu Xinyuan, Bohai Rim Auto City, Tianjin Fenglin Road High School, and Zhujiang Park 18) Area around Tianjin Anding Hospital and Tianjin Medical College 19) Area around Meijiang High School, Wutong Center, and Zuojiangdao Station (Metro Line 1) 20) Area around Tianjin Construction Engineering School, Ruijiang Garden, and Tianjin Auto Parts City 21) Area around Tianjin University of Science and Technology    |
| (3) Nankai District | 1) Area around Tianjin Jinying High School, Nankai District Science and Technology Experimental Primary School, and Tianjin No. 5 High School 2) Area around Pinglu Xili and Nankai District People's Government 3) Area around Guangkai Simaroad Station (Metro Line 2) and Fuxinzhuang Catholic Church 4) Area around the Drum Tower 5) Area around Tianjin World Trade City, Nankai 1911, and Tianjin Nankai Xiangyu School 6) Changhong Ecological Park area 7) Area around Gediao Chuntian, Tianjin No. 50 High School, and Nankai Urban Renewal Memorial Plaza 8) Area around Nankai Park, Xiangyu Park, and Nankai Hospital of Tianjin Integrative Medicine 9) Area around Joy City and Nankai Central Primary School 10) Area around Changjiang Hospital, Yibinli Primary School, and Nankai Affiliated High School of Tianjin Normal University 11) Area around Changzhili Primary School, Prince Zhuang's Mansion, and Fenghe Park 12) Area around Tianjin No. 25 High School, Nankai District Children's Palace, and the First Affiliated Hospital of Tianjin University of Traditional Chinese Medicine 13) Area around the Vocational College of Tianjin Normal University and Tianjin University Beiwucun residential area 14) Area around Lüshuiyuan and Tiantuo Central Park 15) Area around Tianjin Second People's Hospital, Tiankai Plaza, Fenghu Park, and Tianjin First Central Hospital 16) Area around Tianjin University and Nankai University 17) Area around Tianjin No. 63 Center, Tiantuo Hospital, and Wangdingdi Hospital 18) Area around Wangdingdi Station (Metro Line 3), Nanyuan Park, and Binyue Park 19) Area around Tianjin Library, Tianjin Academy of Social Sciences, and Tianjin Administration Institute 20) Area around Tianjin Tennis Center 21) Area around Tianjin Water Park 22) Area around Luneng City Shopping Center and Tianjin Art Vocational College 23) Area around Nancuiping Park 24) Area around Olympic City Commercial Plaza and Tianjin Olympic Center 25) Area around Lingbin Road Station (Metro Line 5) and former Hospital 464 26) Area around Tianjin High School, Tianjin Yan'an Hospital, and Huayuan Civil Air Defense Park    |
| (4) Hedong District | 1) Area around Tianjin Railway Station 2) Area around Tianjin Jiacheng High School and Tianjin Sino-Ocean Future Plaza 3) Area around Wangchuanchang Coach Station and the junior division of Tianjin No. 32 High School 4) Area around Tianjin City Vocational College and Hongcheng 5) Area around Jiahua Dong'an Commercial Plaza and Fengxi Huazhongcheng 6) Area around Jiufuyuan 7) Area around Huaxin Apartments and Tianjin No. 28 High School 8) Area around Jindunli residential community, Hedong District Experimental Primary School, and the University of Technology staff residential compound 9) Area around Tianjin Administrative Licensing Service Center, Tianjin No. 102 High School, and Datong Garden 10) Area around Hedong District No. 2 Central Primary School, Xinghe Garden, and Dongjuzi 11) Area around Qiaoyuan Park, Yihai Shopping Center, and Xiangshan Road Center 12) Area around Tianjin Kerry Centre, Haijin Building, and Hedong Union Hospital 13) Area around Tianzhen Urban Industrial Park and Tianjin Conservatory of Music 14) Area around Tianjin No. 7 High School and Tangkou Park 15) Area around Hedong Park and Wanda Plaza 16) Area around Tianjin Hedong Sports Center and the Tianfei Palace Ruins Museum of the Yuan, Ming and Qing Dynasties 17) Area around Dazhigu Houtai and Tianjin Third Central Hospital 18) Area around Tianjin Aegean Shopping Center and Sixth Avenue 19) Area around Ergong Park 20) Area around Zhongshanmen Park 21) Area around Fumin Park 22) Area around Wanlong Hebin Xinyuan 23) Area around Ningyue Garden, Tianjin No. 1 Commercial School, and Gemdale International Plaza 24) Area around Tianjin No. 98 High School and Lide High School    |
| (5) Hebei District | 1) Area around Wenjinyuan Park, Nanpu Park, and Tianjin First Hospital 2) Area around North Canal Park, Tiantai Road Station (Metro Line 6), and Tianjin Foreign Languages School 3) Area around Shengjing Garden, Huijing Garden, and Zhicheng Zhongli community 4) Area around Xingyi Primary School, Yijing Park, Tianjin No. 30 High School, and Tianjin Academy of Fine Arts (North Campus) 5) Area around Tianjin Fine Arts High School and Tianjin Academy of Fine Arts 6) Area around Joint Logistics Support Force Hospital 983 and Xinkai Primary School 7) Area around Xinkaihe Wanda Plaza, Fulun High School, and Tianjin North Railway Station 8) Area around Beiyu Park 9) Area around Tianjin Railway Technical and Vocational College 10) Area around Siyuan Park 11) Area around Zhongshan Park, Muzhai High School, and Hebei District Experimental Primary School 12) Area around Tianjin Fourth Central Hospital and Tianjin No. 2 High School 13) Area around Hongye Plaza, Tianjin No. 14 High School, and Tianhua Yayuan 14) Area around Hongguang Center and CRCC Plaza 15) Area around Hebei District No. 2 Experimental Primary School 16) Area around Ruihai Mingyuan, Wanghai Gongfu, and Beian Huating 17) Area around Tianjin Italian Style Town 18) Area around Kunwei Road No. 1 Primary School 19) Area around Wangchuanchang Sports Park, Tianjin No. 78 High School, and Vanke City Garden 20) Area around Tianjin Arts and Crafts College and Tongda Xinyuan 21) Area around Emerald Park, Tianjin No. 48 High School, and China Railway Design Corporation 22) Area around the Second Affiliated Hospital of Tianjin University of Traditional Chinese Medicine and Xingfu Park Station (Metro Line 5)    |
| (6) Hongqiao District | 1) Area around Tianjin Chemical Industry School, Tianjin Medical Examination Center, and CNOOC Tianjin Chemical Research and Design Institute 2) Area around Tianjin Third Central Hospital and Hongqiao Hospital 3) Area around Shuixizhuang Park, Hongqiao Park, and the Pingjin Campaign Memorial 4) Area around Tianjin Open University (Hongqiao Branch) and Tianjin School of Finance 5) Area around Hebei University of Technology 6) Area around Kailesai Shopping Center and Hongqiao Primary School Affiliated with Hebei University of Technology 7) Area around Honghuli Station (Metro Line 1) 8) Xiyuzhuang area 9) Area around Xigu Park 10) Area around Shuimu Tiancheng and Longxiyuan 11) Area around Tianjin West Railway Station 12) Area around Qianxiyuan, Tianjin Ethnic Culture Center, and Longyue Garden 13) Area around Red Star Macalline and the Hongqiao Branch of the Tianjin Public Security Bureau 14) Area around Tianjin TEDA Experimental High School and Beikai Garden 15) Area around Tianjin People's Hospital 16) Area around Lüzu Hall 17) Area around Tianjin Fuxing High School and Aqua City (Pengxin) 18) Area around Lujiazui Center and Hebin Garden    |
| (7) Beichen District  Partial area | 1) Area around Taohuasi Xincun 2) Area around Ruijing Street Health and Culture Park 3) Area around Wangzhuang Industrial Park 4) Area around Tianjin University of Commerce 5) Area around Beichen District Secondary Vocational and Technical School 6) Area around Jixian Sports Park and Beichen Hospital 7) Area around Beichen Chaoyang Park and Meiya Building Materials Market 8) Area around Datong Lüdao Jiayuan and Tianjin Hydraulic Press Factory 9) Area around Fangzhou Community, Tianmu Mosque, and the Tianmu Village redevelopment area 10) Area around Beichen Beimen Hospital and Tianjin Nancang High School 11) Area around Danhebei Road Station and Beichentong Station (Metro Line 5) 12) Area around Tianjin Vocational Institute 13) Area around Tasly Health City 14) Area around Zhangxingzhuang Station and Siyuan Road Station (Metro Line 5) 15) Area around Mingzhu Park, Qiyuan Park, and Bichunyuan    |
| (8) Dongli District  Partial area | 1) Area around Yueyahe North Road 2) Area around Haoyangyuan, Lanshanyuan, Haolinyuan, and Ziyuyuan in Sun City 3) Area around Chengcuiyuan, Lüxuanyuan, Danliyuan, Yunliyuan, Xiuliyuan, and Cailiyuan in Sun City 4) Area around the International Community, Kunyu Garden, and Limingzhuang 5) Area around Tianjin Cigarette Factory, Mingzhu Garden, and Xiangyi Plaza 6) Area around the Chinese Armed Police Force Characteristic Medical Center, Tianjin Emergency Medical Center, and Wanshan Road Station (Metro Line 10) 7) Area around Tianjin Furniture Street, Chenglin High School, and Tianjin Public Transport No. 4 Company 8) Area around Shaliu South Road Station (Metro Line 10) and Shangdong Jinmao Smart Science City 9) Area around Tianjin No. 2 Yaohua High School, Sunac City, and Jinyu Yuecheng 10) Area around Tianjin Anning Hospital 11) Area around Dongli Plaza, Dongli High School, and Dongli Park    |
| (9) Jinnan District  Partial area | 1) Area around Tianjin United Family Hospital, Tianyuan Commercial Plaza, and Vanke Crystal City 2) Area around Taihu Road Park and Weijin River Park 3) Area around Meilin Road Station (Metro Line 1), Tianjin No. 2 Xinhua High School, and Tianjin Municipal People's Procuratorate 4) Area around Lushuidao Station (Metro Line 1) and Tianjin Shuanggang High School (Shuanglin Branch) 5) Area around Tianjin University of Technology and Education and Tianjin Haitian Primary School 6) Area around Tianjin Chest Hospital and Tianjin Huanhu Hospital    |
| (10) Xiqing District  Partial area | 1) Area around Xiyingmen Street 2) Area around Qianyuan Industrial Zone and Yingheyuan 3) Area around Bianxing Station (Metro Line 2) 4) Area around Jieyuan West Road Station (Metro Line 2) 5) Area around Dizangwang Temple and Yezi Park 6) Area around Shuixi Park 7) Area around Tianjin Painting Academy 8) Area around Wisdom Hill 9) Area around Sunshine 100 International New City 10) Area around Yaohuan Road Station and Changling Road Station (Metro Line 10), and the First Affiliated Hospital of Tianjin University of Traditional Chinese Medicine (South Campus) 11) Area around Lijiang Road Station (Metro Line 10) and Jiangwan No. 2 Branch Road 12) Area around Meijiang Park and Meijiang Convention and Exhibition Center 13) Area around Meijiang Center Building and Dadao Commercial Plaza    |

5. On this visit, were you seeking medical care yourself or accompanying someone else? ( )

(1) Patient (skip to Question 8)	(2) Accompanying a relative/friend

(3) Paid caregiver (skip to Question 11) (4) Healthcare worker (skip to Question 11)

6. Apart from you, were there any other accompanying persons? If yes, how many? ( )

(1) 1 person	(2) 2 persons	(3) 3 persons	(4) 4 persons	(5) 5 or more persons

7. As an accompanying person, how frequently did you make round trips to the hospital during the preceding three months? ( )

(1) Once only	(2) Once per month	(3) 2-3 times per month

(4) Once per week	(5) 2-3 times per week	(6) Once or more per day

8. Was the patient accompanied on this visit? If yes, by how many people? ( )

(1) No accompanying person	(2) Paid caregiver	(3) One accompanying relative/friend

(4) Multiple relatives/friends accompanying together (5) Multiple relatives/friends taking turns

9. What was the primary reason for the patient's choice of this tertiary Grade-A general hospital? ( )

(1) Strong overall medical capability and reputation (2) Strong specialist department suitable for treatment

(3) Short distance and convenient transportation

10. How many times had the patient visited a healthcare facility during the preceding three months? ( )

(1) First visit	(2) 2-4 times	(3) 5-12 times	(4) 13 or more times

11. What mode of transport did you use for the round trip to the tertiary Grade-A general hospital? ( )

(1) Walking/bicycle/e-bike	(2) Bus	(3) Taxi/private car (4) Metro

12. What time did you leave home? ( )

0-1	1-2	2-3	3-4	4-5	5-6	6-7	7-8

8-9	9-10	10-11	11-12	12-13	13-14	14-15	15-16

16-17	17-18	18-19	19-20	20-21	21-22	22-23	23-24

13. What is your usual travel time to this hospital? ( )

(1) <0.5 h (2) 0.5-1 h (3) 1-1.5 h (4) 1.5-2 h (5) >=2 h

14. How much time do you usually spend in traffic congestion on the way to the hospital? ( )

(1) 0-15 min	(2) 15-30 min	(3) 30-60 min

(4) 60-120 min	(5) More than 120 min

15. Under which of the following circumstances would you prefer to travel to and from the tertiary Grade-A general hospital by taxi or private car? ( )

(1) Severity of illness	(2) Excessive travel distance or time

(3) Proximity to an expressway, allowing rapid access despite the long distance (4) Inconvenient public transport

Note: This question was displayed only when walking/bicycle/e-bike, bus, or metro was selected in Question 11.

16. If bus services provided a more convenient and faster journey to this tertiary Grade-A general hospital, under which condition would you prefer to travel by bus? ( )

| Travel arrangement/number of stops | Within 5 stops | Within 10 stops | Within 15 stops | More than 15 stops | Not acceptable |
| --- | --- | --- | --- | --- | --- |
| Direct bus service | A | B | C | D | M |
| One transfer | E | F | G | H | M |
| Two or more transfers | I | J | K | L | M |

Note: This question was displayed only when taxi/private car was selected in Question 11.

17. What travel distance to a tertiary Grade-A general hospital would you usually consider acceptable? ( )

(1) 0-3 km (2) 3-5 km (3) 5-10 km (4) 10-20 km (5) More than 20 km

18. What travel time to a tertiary Grade-A general hospital would you usually consider acceptable? ( )

(1) 0-15 min	(2) 15-30 min	(3) 30-60 min

(4) 60-120 min	(5) More than 120 min

19. Under otherwise equivalent medical conditions, why would you choose a tertiary Grade-A general hospital? ( )

(1) More beds and a larger hospital scale	(2) Shorter distance	(3) Convenient parking

(4) Convenient public transport (5) Clearly organized outpatient, emergency, diagnostic/technical, and inpatient services

Note: Multiple responses were permitted.
